# Coexisting Angiomyolipoma and Renal Cell Carcinoma in a Kidney of an Elderly Woman: Case Report and Review of the Literature

**DOI:** 10.1100/tsw.2004.41

**Published:** 2004-06-07

**Authors:** Barry Billings, Leon C. Hamrick, Anton J. Bueschen, Philip J. Kenney

**Affiliations:** Division of Urology, University of Alabama at Birmingham and Saint Vincents Hospital, Birmingham, AL, USA

## Abstract

Angiomyolipoma is a well described but relatively uncommon benign renal neoplasm composed of varying admixtures of mature adipose tissue, smooth muscle, and thick-walled blood vessels.[1] The incidence of angiomyolipoma is about 0.3% overall.[2] It frequently occurs in patients with tuberous sclerosis. Even more uncommon is the simultaneous occurrence of angiomyolipoma and renal cell cancer in the same kidney in a patient without tuberous sclerosis.

## CASE REPORT

An 86 year-old woman presented to the emergency room with acute onset of nausea and vomiting associated with right lower quadrant abdominal pain. History was significant for a fall one week earlier. Physical exam showed moderate right-sided abdominal fullness and tenderness. Hematocrit was 25 and urinalysis was negative. Abdominal CT showed two space-occupying right renal masses (Figs 1,2). The upper poles mass appeared solid, while the lower pole showed a larger fat containing heterogeneous mass with perirenal hemorrhage and inflammatory changes. Renal ultrasound confirmed the solid nature of the upper pole mass (Fig 3). The following day, the patient underwent a radical nephrectomy due to intractable pain and unstable hemorrhage requiring the transfusion of two units of red blood cells.

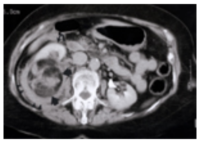

**FIGURE 1**. Contrast enhanced CT shows fat containing mass (arrows) arising from mid portion right kidney (note residual parenchyma (curved arrows)). Soft tissue density strands surround the mass and the kidney, typical of hemorrhage.

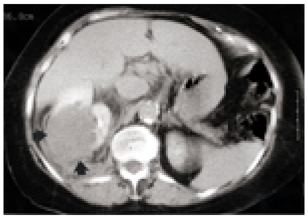

**FIGURE 2**. Contrast enhanced CT of the upper pole reveals a solid appearing mass (arrows).

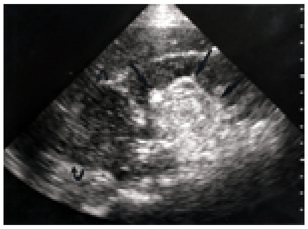

**FIGURE 3**. Longitudinal sonogram of the right kidney confirms the presence of an isoechoic mass of the upper pole (curved arrows) and a seperate echogenic mass (arrows) in mid and lower pole.

Pathologic examination demonstrated the upper pole tumor to be a 7 cm well differentiated clear cell carcinoma which did not penetrate the renal capsule. The larger lower pole tumor, measuring 9.5 x 8.5 x 7.5 cm, was a ruptured angiomyolipoma. This lesion had a characteristic pattern of tortuous large blood vessels within the tumor composed primarily of mature fat. Surrounding the vessels, there was sparse smooth muscle showing cells which had characteristic moderate atypia and an epithelioid appearance. The hemorrhage in the perirenal fat was associated with the ruptured angiomyolipoma.

Convalescence was normal.

## DISCUSSION

The coexistence of an angiomyolipoma and a renal cell carcinoma is very rare. The first recorded case in the literature was in 1975 by Kavaney and Fielding.[3] A review of the literature has elicited reports of only 31 similar cases. Of the 31 cases, 17 have been in patients with tuberous sclerosis, and only 14 in nontuberous sclerosis patients.[4] Coincident angiomyolipomas and renal cell cancers have been found to be mixed in the same tumor mass or my be entirely separate masses, as in this patient. The patients without tuberous sclerosis were between 39 and 74 years old, with mean age of only 53 years old, while those with tuberous sclerosis were between 11 and 57 years old with a mean age of 31 years of age.[4] Our 86 year old patient was much older.

Angiomyolipoma, although rare, is the most common benign renal neoplasm. The clinical presentation of the neoplasm varies with its size. Smaller lesions, those defined as being less than 4 cm., can present with microhematuria or chronic mild flank pain. However, with current imaging techniques, asymptomatic angiomyolipomas are often discovered incidentally by CT or Ultrasonography. The second most common presentation is spontaneous hemorrhage, either intratumoral or perinephric. Larger lesions, those greater than 4 cm., are often symptomatic and may present with hematuria, shock, intractable pain, palpable mass, or acute hemorrhage, either intratumoral or into the retroperitoneal space.[2,5] Mouded et al reviewed the symptomatology in 97 patients with angiomyolipoma and found that 15% presented with spontaneous retroperitoneal hemorrhage.[6] Oesterling et al reviewed 602 patients with renal angiomyolipoma from the world literature, and found that 51% with angiomyolipomas larger than 4 cm. presented with spontaneous retroperitoneal hemorrhage and 9% presented with shock.[7]

Renal cell carcinoma is the most common solid renal tumor and is the most common malignant tumor associated with retroperitoneal hemorrhage. However, overall, this is a relatively uncommon presentation. Skinner et al found only 1 presentation of retroperitoneal hemorrhage in a series of 329 patients with renal carcinoma.[7] Chang et al reported on a series involving 146 patients where only 2 presented with retroperitoneal hemorrhage.[8]

The hallmark of angiomyolipoma on CT is the presence of fat. Negative attenuation values of -15 HU to -100 HU are commonly seen which are indicative of adipose tissue. The neoplasm may have varying admixtures of fat and smooth muscle, but is rarely mostly solid. Calcifications are rarely seen in the tumors. With ultrasonography, angiomyolipomas are the most echogenic renal neoplasm.[2]

Histopathologically, an angiomyolipoma is a choristoma composed of mixtures of smooth muscle, blood vessels, and adipose tissue, often in haphazard fashion. The term choristoma is correct, as fat is not a normal component of renal parenchyma. Pleomorphism is common within the tumor, however, mitotic figures are rarely seen. There is frequentaly evidence of hemorrhage, especially in symptomatic cases. The blood vessels seen within the tumor are usually quite abnormal. They are thick-walled with generally absent internal elastic laminae, often tortuous, and frequently in an angiomatous arrangement.[2] With its degree of vascularity as well as the tortuous and rigid nature of the blood vessel walls, angiomyolipomas are thus quite prone to hemmorhage.

Our patient presented with pain, but her primary complaint was that of acute onset of nausea and vomiting. On laboratory analysis, she had anemia, but she did not present with shock. The initial CT was somewhat confusing. There was evidence of hemorrhage, a fat containing mass, and a non fat containing mass. The differential diagnosis was a complex hemorrhagic renal mass, and a non fat containing mass. The differential diagnosis was a complex hemorrhagic renal mass suspicious for renal cell carcinoma or angiomyolipoma versus an inflammatory mass or possible renal abscess.

It was the final pathology which confirmed the true diagnosis of these renal masses. Our patient did indeed have a large upper pole renal cell cancer confined within the renal capsule. However, it was the larger lower pole angiomyolipoma which had ruptured both, within the tumor and into the perinephric space, that initiated the patient's presentation.
